# USING THE PLATFORM OF A NATIONAL CENTER TO INTEGRATE RESEARCH, PRACTICE, AND POLICY: CEAL@UNC

**DOI:** 10.1093/geroni/igae098.1028

**Published:** 2024-12-31

**Authors:** Sheryl Zimmerman

**Affiliations:** University of North Carolina at Chapel Hill (UNC), Chapel Hill, North Carolina, United States

## Abstract

The intent of research is to inform practice and policy to benefit society. Unfortunately, just as academic units are often siloed, so too are organizations constituting the three-legged stool of research, practice, and policy that must work together toward the greater good. One strategy to promote the intersection of the three is the platform of a national center, such as the Center for Excellence in Assisted Living (CEAL). Assisted living is the largest provider of residential long-term care in the nation, the importance of which was recognized in 2001 by the US Senate Special Committee on Aging and led to the development of CEAL to promote quality in assisted living. For 20 years, CEAL thrived as a unique collaborative of diverse national organizations, and in 2023 joined with the University of North Carolina at Chapel Hill (CEAL@UNC) to expand capacity to develop evidence, bolster the workforce, and promote the adoption of evidence-based practices and policies. CEAL@UNC is exploring and implementing new avenues to bring together individuals and organizations representing different constituencies (e.g., the development of national consumer, practice, policy, and research cores), and capitalizing on its broad representation to inform efforts at numerous levels, including the federal government. This session will discuss the evolution of CEAL to CEAL@UNC, the process of strategic planning and priority setting, and strategies to become a consolidated voice for the intersection of research, practice, and policy – all of which are relevant for topics other than assisted living.

